# Study of the Microstructure and Corrosion Properties of a Ni-Based Alloy Coating Deposited onto the Surface of Ductile Cast Iron Using High-Speed Laser Cladding

**DOI:** 10.3390/ma15051643

**Published:** 2022-02-22

**Authors:** Rui Wang, Changyao Ouyang, Qihang Li, Qiaofeng Bai, Chunjiang Zhao, Yingliang Liu

**Affiliations:** 1 School of Mechanical Engineering, Taiyuan University of Science and Technology, Taiyuan 030024, China; b20180011@stu.tyust.edu.cn (R.W.); s20190160@stu.tyust.edu.cn (C.O.); s20201201013@stu.tyust.edu.cn (Q.L.); newmaker@tyust.edu.cn (Q.B.); 2 School of Mechanical and Equipment Engineering, Hebei University of Engineering, Handan 056038, China; 3 Shuozhou Jinhua Industrial Co., Ltd., Shuozhou 036000, China; liuyang123vi@126.com

**Keywords:** laser power, ductile cast iron, Ni-based alloy coating, corrosion performance, corrosion mechanism

## Abstract

To improve the surface corrosion resistance of ductile iron, Ni-based alloy coatings were prepared using a high-speed laser cladding technology with different levels of laser power. The microstructure, phases, and corrosion properties of the coatings were investigated by scanning electron microscopy (SEM), X-ray diffraction (XRD), and an electrochemical workstation. Variations in laser power did not change the main phases of the coatings, which were composed of γ-Ni, Ni_3_B, Ni_2_Si, and Cr_23_C_6_. With an increase in power, the degree of segregation in the coating decreased, sufficient melting between elements was achieved, and the chemical composition became more uniform. Enhancement of the laser power resulted in more energy being injected into the cladding, which allowed adequate growth of tissue, and dendrites continued to grow in size as the power increased. The self-corrosion potentials of the coatings at laser power levels of 1.6, 2.0, and 2.4 kW were −625.7, −526.5, and −335.7 mV, respectively. The corrosion potential of the 2.4 kW coating was the highest, and the corroded surface of the cladding layer included mainly sizeable continuous structures with a light degree of corrosion and the highest corrosion resistance.

## 1. Introduction

Cast iron is widely used in industrial production because of its good castability and fatigue resistance, and its other advantages [1]. Because its mechanical properties are similar to those of steel, ductile cast iron is used to manufacture parts with long lifespans, large volumes, and complex shapes, subjected to complicated forces, such as camshafts, crankshafts, cylinder blocks, valve bodies, etc. [2,3,4]. Furthermore, ductile iron parts are frequently used in harsh working environments involving factors such as wear, corrosion, and stress alternation, which cause failure and damage. To further improve the service life of ductile iron parts, the surface of damaged ductile iron parts can be repaired. Moreover, surface modification and strengthening of ductile iron parts can also improve their service life in harsh working environments [5,6]. Currently, laser cladding technology is extensively applied in material failure repair and surface modification. It not only ensures full utilization of the properties of the cladding materials but also preserves the mechanical properties of the substrate, in addition to having the advantages of low cost, high speed, and low pollution [7,8].

Li et al. [9] prepared a Fe–Ni–Cr alloy coating on the surface of ductile iron, and the hardness and wear resistance of the cladding layer were significantly improved. Liu et al. [10] used Inconel 625 superalloy as the transition layer on the surface of ductile cast iron and an SS420 stainless steel working layer was prepared using laser deposition. The surface hardness of the coating was about 2.5–3 times the hardness of the substrate. Zhu et al. [11] prepared a Fe–Cr alloy coating on ductile iron laser cladding to improve the hardness and wear resistance of the substrate. Sun et al. [12] obtained a wear-resistant layer on a ductile iron roll containing C–B–W–Cr powder, using laser surface alloying technology. Zhang et al. [13] prepared iron-based cladding and nitride layers on a ductile iron surface to guide the remanufacturing of ductile iron. Li et al. [14] performed laser cladding of ductile iron using a powder embedding method, resulting in significantly less dilution and elimination of the cold structure, and the microhardness characteristics were investigated. Li et al. [15] coated Ni–Cu-based alloy powders on the surface of ductile iron and studied the relative phase evolution, migration of different elements near the interfacial zone, partial melting during single-pass and multi-layer cladding processes, and heat-affected microhardness. However, current studies of laser cladding on ductile iron surfaces have mainly investigated its hardness and wear resistance, whereas its corrosion performance has been less widely studied.

The metal Ni has medium hardness and good malleability, and it can maintain stable plasticity at high temperatures, which hinders the diffusion of carbon elements within the substrate into the cladding layer during the cladding process [16]. At the same time, Ni-based alloys have good corrosion resistance, so they are often used on components requiring high corrosive resistance. With the aim of applying ductile cast iron in adverse working environments resulting in severe corrosion, and in light of the excellent performance of Ni-based alloys, laser cladding was used to prepare a Ni-based alloy coating on the surface of ductile iron to improve its corrosion resistance. Currently, there is little research on enhancing the corrosion resistance of ductile cast iron via laser cladding of its surface with Ni-based alloys. Therefore, it is important to study whether this strategy can be used to improve the corrosion resistance of ductile cast iron. The selection and matching of the processing parameters used during laser cladding are closely related to the quality, structure, and properties of the resulting coating, and the laser power is one of the typical process parameters that is closely linked [17].

High-speed laser cladding has been widely applied in actual production and repair due to its advantages of high speed, high efficiency [18], and less dilution [19]. To improve the corrosion resistance of the surface of ductile cast iron, high-speed laser cladding technology was used in this study to prepare a Ni-based alloy coating with a high Ni content on the surface of ductile cast iron, using varying levels of laser power. The influence of laser power on the microstructure, phase, and corrosion resistance of the resulting cladding layer was investigated. The results provide a reference for cladding Ni-based alloy coatings on the surface of ductile cast iron to improve its corrosion resistance.

## 2. Materials and Methods

### 2.1. Material

**Figure 1:**
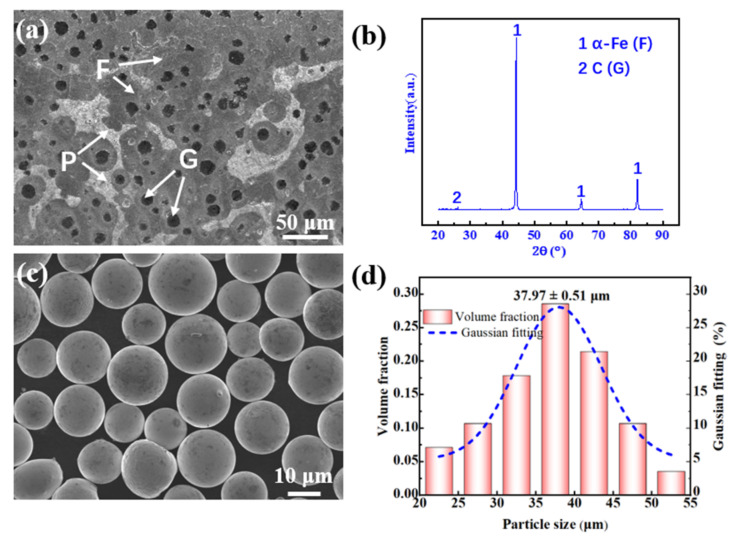
Ductile cast iron and Ni-based alloy powders: (**a**) ductile cast iron; (**b**) substrate XRD spectrum; (**c**) powder morphology; (**d**) the particle size distribution.

A ductile iron plate (210 mm × 210 mm × 20 mm in size) was used as the cladding substrate. Prior to cladding, a grinder was used to grind and polish the designated cladding area to remove substrate surface rust and dirt. The microstructure of the ductile cast iron matrix was composed of ferrite (F) and pearlite (P), while the spherical graphite (G) was surrounded by ferrite (Figure 1a). X-ray diffraction (Figure 1b) showed that the substrate was mainly composed of a ferrite phase (α-Fe) and a graphite phase (C). The cladding powder was a spherical Ni-based alloy (approximately 95 wt.% Ni content, Figure 1c), with an average particle size of approximately 37.97 μm (Figure 1d) and good flow properties. The compositions of the substrate and powder are listed in Table 1.

### 2.2. Laser Cladding Process

**Figure 2:**
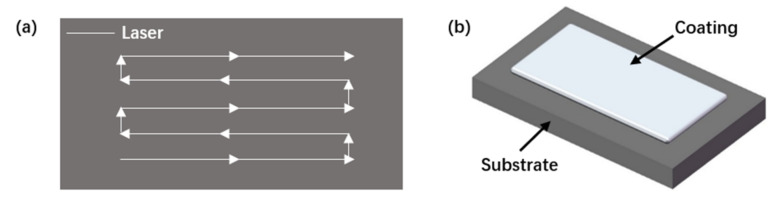
Schematics of: (**a**) the laser scanning pattern; (**b**) sample preparation.

An RFL-C4000 (Raycus Fiber Laser, Wuhan, China) high-power fiber laser (rated power: 4.0 kW; wavelength: 1080 ± 5 nm) was applied to drive the laser cladding head with a KUKA manipulator to realize the change in spatial displacement. A model DPSF-2 powder feeder (Dual Package System Framework, Shanghai, China) was used (the protective gas and powder feed gas were Ar), and laser cladding with synchronous powder feeding was applied to the substrate. The laser scanning track is shown in Figure 2a for the application of the coating in a single layer (Figure 2b).

**Figure 3:**
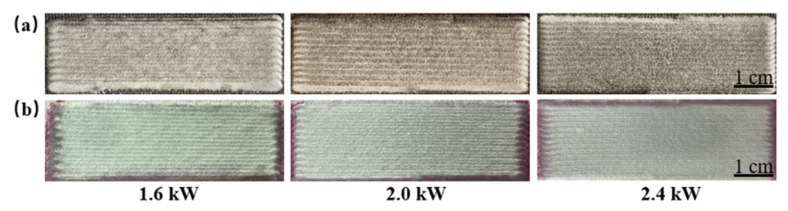
Images showing (**a**) the surface morphology of coatings and (**b**) crack detection results for cladding layers prepared using different laser powers of 1.6, 2.0, and 2.4 kW as indicated.

After completing the experimental studies, we selected an intact surface with satisfactory molding and no apparent defects as the standard to determine the process parameters. The optimized parameters for the laser cladding are listed in Table 2. Figure 3 shows images of the cladding layer prepared at 1.6, 2.0, and 2.4 kW. The visible surface was intact, and no cracks were found after flaw detection. The laser energy density (LED) was calculated according to Equation (1) [20]:

(1) $$LED=\frac{P}{\pi \times {\left(D/2\right)}^{2}\times v}\;$$

where *P* is the laser power, *D* is a laser spot diameter of 2.5 mm, and *v* is the scanning speed.

### 2.3. Test Method

A cross section of the cladding layer sample was extracted using a wire cutting machine and polished, and aqua regia (HCl/HNO_3_ = 3:1) was then used to etch the coating for 8–15 s. A field emission scanning electron microscope (SEM, JSF-7001) was used to observe the microstructure and the corrosion morphology of the sample. Elemental composition tests were conducted by energy-dispersive spectrometry (EDS) using a Bruker XTrace. Electron backscatter diffraction (EBSD, Oxford Nordlys Max3) and Empyrean X-ray diffraction (XRD, λ = 0.154060 nm, step size = 0.026°) were used to characterize the coating phases. A three-electrode RST5000 electrochemical workstation was used to test the corrosion resistance of the samples. In the electrochemical corrosion test, 3.5 wt.% NaCl was used as the electrolyte solution with a saturated calomel reference electrode and Pt as the auxiliary electrode. The sample was polished, wiped with alcohol, and sealed with hot-melt adhesive, leaving only the cladding layer (area 1 cm^2^) as the working electrode. During the impedance spectrum test, harmonic signals with an amplitude of 10 mV and a frequency range of 10^5^~10^−^^2^ Hz were applied. The polarization curve was measured using a potentiostatic dynamic measurement with a scan rate of 0.5 mV/s and a voltage range from −1.0 to 0.8 V. To improve the measurement accuracy, the procedure was carried out three times under the same experimental conditions, and the measurement results were averaged.

## 3. Results and Discussion

### 3.1. XRD Phase Analysis

**Figure 4:**
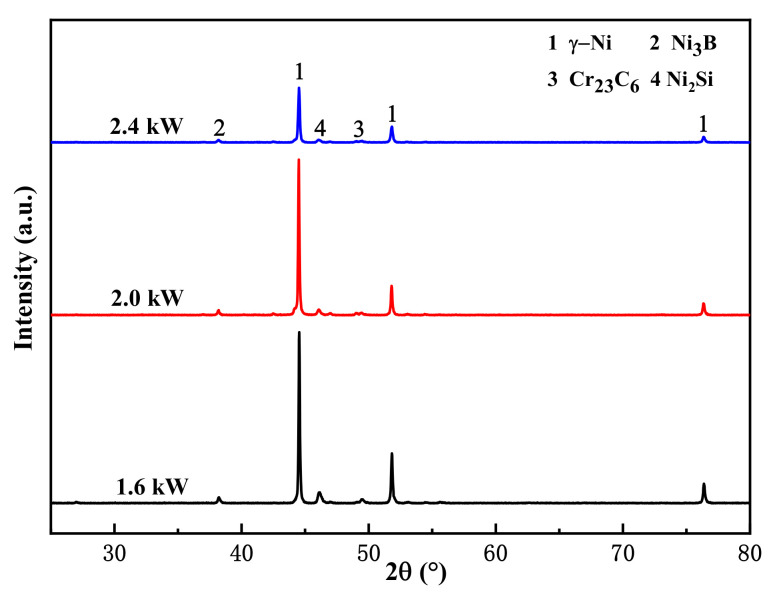
XRD pattern of the Ni-based alloy coating.

Figure 4 shows the phases of the Ni-based alloy coatings prepared under three levels of laser power. The coating was mainly composed of solid solution γ-Ni (PDF#87-0712), a boride Ni_3_B phase (PDF#73-1792), a silicide Ni_2_Si phase (PDF#73-2093), and a carbide Cr_23_C6 phase (PDF#71-0552). The three main peaks of the coating (2θ = 44.5°, 51.8°, and 76.4°) were mainly solid solution γ-Ni, which belongs to the Fm-3m space group, and the three diffraction peaks correspond to the (111), (200), and (220) crystal planes. During laser cladding, Ni, B, and Si elements in the high-temperature molten pool could form nickel boride and nickel silicide, and Cr and C could form chromium carbide [21,22,23].

3Ni + B → Ni_3_B (2)

23Cr + 6C → Cr_23_C_6_ (3)

2Ni + Si → Ni_2_Si (4)

The peak positions of the diffraction peaks of the three coatings were identical, and no new diffraction peaks were found, which indicates that there was no change in the main phases of the coatings prepared using different levels of laser power. Because the heat input variation ranges of the three samples were similar, the thermodynamic conditions were approximately similar [24,25]. According to previous studies [26], other researchers obtained similar conclusions when studying laser power on 17-4PH stainless steel coatings.

### 3.2. Microstructures

#### 3.2.1. Surface Microstructure

**Figure 5:**
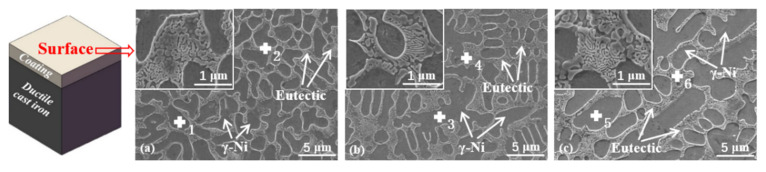
Microstructure of the coating surface prepared with laser powers of (**a**) 1.6 kW, (**b**) 2.0 kW, and (**c**) 2.4 kW.

**Figure 6:**
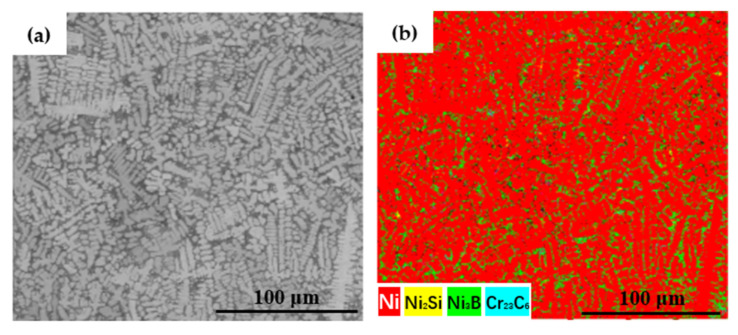
EBSD phase map of eutectic structure and the solid solution of the 2.4 kW coating: (**a**) microstructure distribution; (**b**) EBSD phase distribution.

Figure 5 shows images of the coating surface microstructure. The microstructure of the cladding layer shows crystallization in the shape of dendrites and was mainly composed of a bright white eutectic structure and a dark-gray primary solid solution phase. The eutectic structure mainly occurred between the dendrites, and the primary solid solution occurred in the dendrites. The eutectic structure between the dendrites was looser after cladding at 1.6 kW (Figure 5a) and was relatively dense between the dendrites in the samples after cladding at 2.0 and 2.4 kW (Figure 5b,c). In the EBSD phase map (Figure 6), the primary phase of the coating was mainly a γ-Ni solid solution, and the eutectic structure was mainly composed of the Ni_3_B phase and the Ni_2_Si and Cr_23_C_6_ phases. The eutectic structure of the phase diagram was mainly dominated by the green Ni_3_B phase and yellow Ni_2_Si phase, indicating that the eutectic structure contained a large amount of Ni_3_B and Ni_2_Si. The color of the Cr_23_C_6_ phase was mixed or masked, so it was not apparent. The coating structure was further analyzed, considering also the results for the elemental content revealed by EDS in Table 3. The contents of B, C, and Si in the eutectic structure region in the 1.6 kW coating were much higher than those in the solid solution structure. This is because non-metallic elements are easily enriched and readily precipitate in the interdendritic region, which led to the segregation of B, C, and Si in the interdendritic region to form a eutectic structure. The eutectic structure appeared in lamellar form through eutectic solidification at the eutectic temperature [27]. In a previous study [28] of the effect of chemical segregation on nanobainitic transformation in laser cladding coatings, the positive segregation of Si elements in the interdendritic region was reported. In another study [24] on the effect of laser scanning speed on the microstructure of a T15M coating prepared by laser cladding, it was reported that the segregation of C in the interdendritic region was more severe. Similar conclusions were also obtained in our study of a Ni-based alloy coating, where the element segregation phenomenon weakened with the increase in laser power. Less segregation was observed in the 2.4 kW coating, which contained a large amount of C in both the eutectic structure and the solid solution. The higher laser energy input may have accelerated the diffusion of C and caused more elements to solid-dissolve into γ-Ni, resulting in strengthening of the solid solution. As the power increased, the components of the coating were melted more completely and the convection intensity in the laser molten pool was also higher; these conditions are more conducive to achieving component uniformity [29].

#### 3.2.2. Section Microstructure

**Figure 7:**
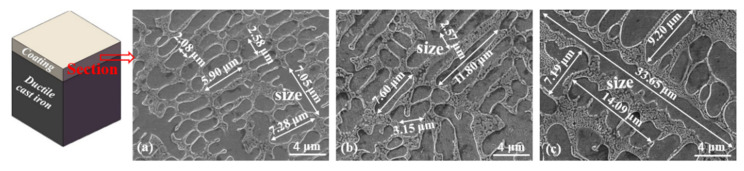
Microstructure cross section of the coatings prepared using laser powers of (**a**) 1.6 kW, (**b**) 2.0 kW, and (**c**) 2.4 kW.

Figure 7 displays the cross-sectional microstructure of the coating. Compared with the microstructure of the coating prepared under 2.0 and 2.4 kW, the cross-sectional microstructure of the 1.6 kW cladding layer was denser, relatively fine, and more uniform (Figure 7a). The microstructure particle size of the coating section of the 2.0 kW sample was larger (Figure 7b), while that of the 2.4 kW sample was the largest, especially in the long-axis dendrite region (Figure 7c). The size of the microstructure increased with the laser power. As reported by Wan et al. [30], the laser power determines the amount of energy injected into the cladding layer by the laser beam, i.e., the size of the LED. The LED energy at 1.6, 2.0, and 2.4 kW was 10.86, 13.58, and 16.30 J/mm^3^, as shown in Table 2. The 2.4 kW laser power provided greater energy density, which increased the heat input into the cladding layer, increased the temperature of the molten pool, and prolonged the duration of the molten pool. Under other conditions, it was observed that the higher the laser power, the greater the energy injected into the cladding layer, the slower the solidification rate, and the larger and sparser the microstructure particles [31].

**Figure 8:**
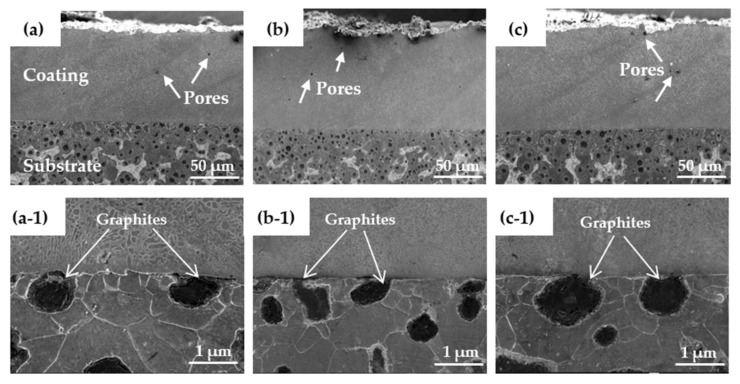
The interface morphology of the coating and substrate for laser cladding with laser powers of (**a**,**a**-**1**) 1.6 kW, (**b**,**b**-**1**) 2.0 kW, and (**c**,**c**-**1**) 2.4 kW.

Figure 8 shows the interface morphology of the coating and the substrate. The overall integrity of the cladding layer had no cracks or obvious defects, and there was a small number of pores. Compared with ordinary laser cladding [32], the entire bonding line corresponding to the interface between the high-speed laser cladding coating and the substrate was relatively straight. Therefore, at the interface, few differences were observed between coatings prepared using the three different laser power levels. This was mainly due to the characteristics of rapid cladding and solidification of high-speed laser cladding, which resulted in a Ni-based alloy coating that was less diluted at the interface. It can be seen in Figure 8 that at the interface of the coating, only a small amount of carbon in the graphite melted into the coating, which indicates that less carbon from the ductile iron entered the cladding layer. Because the instantaneous energy of the laser cladding was high and concentrated, its surface melted when it came into contact with the ductile cast material, and the melted nodular material entered the cladding layer through the convective mass transfer of the molten pool [33].

### 3.3. Electrochemical Corrosion

**Figure 9:**
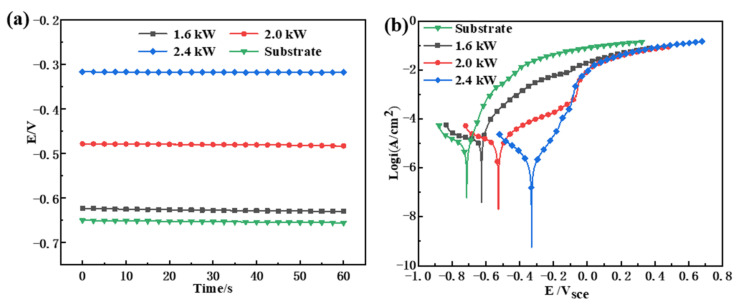
(**a**) OCP diagram and (**b**) polarization curves for substrate and coatings for laser cladding using different laser powers.

The open circuit potential (OCP) and polarization curves of the substrate and the cladding layer are illustrated in Figure 9. The OCP of the cladding layers is displayed in Figure 9a. The system remained stable after the transient process, and the anodic and cathodic reactions reached a state of relative equilibrium [34]. In a previous study [35], it was shown that the higher the OCP, the lower the possibility of corrosion tendencies. Wong et al. [36] also obtained similar electrochemical corrosion results for laser alloy samples, i.e., indicating that the OCPs of the coatings prepared with different laser power levels were higher than that of the substrate, indicating that the corrosion tendency of the coating was lower than that of the substrate. Compared with the coatings prepared at 1.6 and 2.0 kW, the cladding layers prepared under 2.4 kW had the highest corrosion resistance, as reflected by the higher OCP. Due to the increase in laser power, the shielding effect and thermodynamic stability of the coating against corrosive ions were improved [37]. Meanwhile, it was impossible to judge the rate and specific situation of the corrosion process. As a result, it is necessary to conduct a more detailed electrochemical test and analysis of the corrosion of the cladding layer.

Figure 9b exhibits the potentiodynamic polarization curves of the cladding layers fabricated with different levels of laser power. The three coatings have similar polarization processes. As shown in Table 4, according to the Tafel extrapolation method, the self-corrosion potential *E_corr_* of the substrate was −713.5 mV, and the self-corrosion potentials at 1.6, 2.0, and 2.4 kW were −625.7, −526.5, and −335.7 mV, respectively. The *E_corr_* was measured when an AC harmonic signal was applied to the system, so the value is different from the OCP. The *E_corr_* reflects the resistance of the material to corrosion. The lower the corrosion potential, the higher the corrosion tendency of the material [38]. The current exchange densities of the anodic reaction and cathodic reaction (i_a_ and i_c_) were linearly correlated with the self-corrosion current, *i_corr_* [38]. Meanwhile, under the same conditions, *i_corr_* reflects the corrosion rate, as calculated by Equation (5), and the smaller the value, the higher the corrosion resistance of the material.

(5) $$v=\frac{M}{nF}i_{corr}$$

where v is the corrosion speed (g/m^2^ h), *i_corr_* is the corrosion current density (μA/cm^2^), *M* is the atomic weight of the metal (g), *n* is the valence of the metal, and *F* is Faraday’s constant.

The rankings of *E_corr_* and *i_corr_* are:

$$E_{corr}(\mathrm{Substrate})<\;E_{corr}(1.6\;\mathrm{kW})<\;E_{corr}\left(2.0\;\mathrm{kW})\;<E_{corr}(2.4\;\mathrm{kW}\right)\;i_{corr}(\mathrm{Substrate})>\;i_{corr}(1.6\;\mathrm{kW})>i_{corr}\left(2.0\;\mathrm{kW})\;>i_{corr}(2.4\;\mathrm{kW}\right)$$

When the laser power was 2.4 kW, the $E_{corr}$ of the cladding layer was the highest, the *i_corr_* was the smallest, the corrosion rate was the slowest, and the corrosion resistance was higher. According to this analysis, an increase in laser power resulted in improved corrosion resistance of the resulting cladding layer. At the same time, by fitting the measurement results of the electrochemical workstation system, it was found that the annual average corrosion rate of the 2.4 kW coating was 0.00699 mm·y^−^^1^, which is much smaller than those of the 2.0 and 1.6 kW coatings.

**Figure 10:**
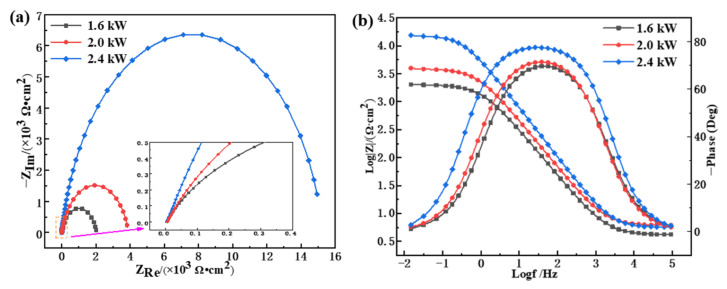
EIS diagram of the cladding layers with different levels of laser power: (**a**) Nyquist; (**b**) Bode.

Figure 10 shows the electrochemical impedance spectroscopy (EIS) images of the cladding layers prepared with different laser power levels. The Nyquist curves of the cladding layer all deviated from a semicircle, with similar variation trends (Figure 10a). The length of the line between any point on the Nyquist curve and the origin point was used to express the impedance value |*Z*|, and the angle between the straight line and the horizontal axis represents the impedance angle (φ), as given in Equation (6). When the frequency changed from 100 kHz to 0.01 Hz, the cladding layers at different power levels showed prominent characteristics of capacitive reactance, the Nyquist locus transformed from high frequency (close to the origin) to low frequency (away from the origin point), and the |*Z*| value gradually increased. Previous studies [39,40] found that a larger capacitive arc radius is indicative of higher cladding surface corrosion resistance. In this study, the 2.4 kW cladding layer possessed the largest arc radius and impedance value and the highest corrosion resistance, while the 1.6 kW coating had the lowest corrosion resistance.

(6) Z=ZRe2+ZIm2φ=arctan−1ZImZRe

where $Z_{Re}$ and $Z_{Im}$ are the real and imaginary parts of the impedance spectrum. When harmonic signals of the same frequency are applied in an electrochemical system, the impedance spectrum characteristics corresponding to cladding layers with different levels of laser power can be quantitatively described using a Bode diagram, as shown in Figure 10b. At low frequency, the impedance of the 2.4 kW cladding layer (the maximum value was approximately 17778.79 Ω·cm^2^) was nearly 10 times higher than that of the 1.6 kW cladding layer (the maximum was about 1995.26 Ω·cm^2^), and the corrosion resistance was greatly improved. Low-and middle-frequency |Z| was approximately linearly correlated with frequency. The maximum phase angle was between 70° and 80°, and the phase angle deviation from the ideal capacitance was minor, which indicates strong capacitance characteristics. This is consistent with the properties indicated by the Nyquist diagrams. According to Figure 10b, the corresponding impedance value and phase angle of the cladding layer with the same frequency was greater at 2.4 kW than at 1.6 and 2.0 kW, and the 2.4 kW cladding layer had high corrosion resistance.

**Figure 11:**
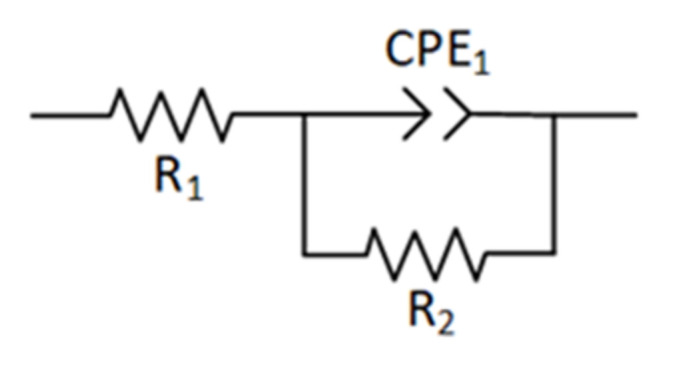
Equivalent circuit of the coatings.

Circuit fitting was carried out according to the impedance spectrum to understand the electrochemical performance of cladding layers formed by different laser power levels. The phase-frequency diagram shown in Figure 10b has a single “hump”, indicating that the electrochemical system was constant at one time point, where the corrosion process only corroded the cladding layer surface but did not erode the substrate through the coating. An equivalent circuit is illustrated in Figure 11, where *R*_1_ represents the solution resistance between the working electrode and the reference electrode. The impedance of the charged particles passing through a double layer is expressed as *R*_2_. The interface’s double-layer capacitance between the solution and the cladding layer and the capacitance caused by the surface inhomogeneity can be represented using the constant phase element CPE_1_ [40]. The impedance value is corrected via Equation (7).

(7) $$\left\{\begin{array}{l} \left|Z_{CPE}\right|=\frac{\omega^{-n}}{Q_{0}}\\ \varphi =\frac{n\pi }{2} \end{array}\right.$$

where *Q*_0_ is the capacitance of CPE_1_, and *n* is the deviation from the ideal capacitance. The value of *n* reflects the strength of the dispersion effect in the electrochemical reaction process. If the numerical value of *n* is closer to 1, the deviation from the ideal capacitance is minor and the dispersion effect is weaker. The roughness of the working electrode surface and the uneven distribution of the corrosion current density are related to *n*. The total impedance of the equivalent circuit is expressed using Equation (8), where *Z_T_* is the total impedance, *Z_F_* is the admittance corresponding to the parallel branch of *R*_2_ and CPE_1_, and *Y_F_* is the admittance of the parallel branch.

(8) $$\left\{\begin{array}{l} Z_{T}=R_{1}+Z_{F}\\ Z_{F}=\frac{1}{Y_{F}}\\ Y_{F}=\frac{1}{R_{2}}+Q_{0}\omega^{n}\left(\cos\frac{n\pi }{2}+j\sin\frac{n\pi }{2}\right) \end{array}\right.$$

According to the electrochemical impedance spectra (EIS), the impedance data obtained by ZView fitting are listed in Table 5. When the laser power increased from 1.6 to 2.4 kW, the *R*_2_ value increased significantly, and the *n* value was slightly enhanced, while CPE_1_ decreased. The change in n was consistent with the linear correlation between impedance and frequency within a particular range, as shown in Figure 10b. When the power was constant, *R*_2_ was much larger than *R*_1_, showing that the migration of charged particles played a dominant role in the corrosion process of the working electrode. The *R*_2_ value of 2.4 kW was maximal, indicating maximized particle migration resistance and better corrosion resistance. This conclusion is consistent with the analysis of the dynamic potential polarization curve and impedance spectra. The cladding layer surface was more uniform when the laser power was higher, with fewer defects and better corrosion resistance.

### 3.4. Corrosion Mechanism

**Figure 12:**
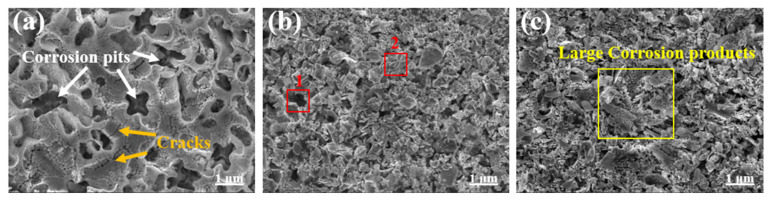
Corrosion morphology after a polarization test of the coatings prepared using laser powers of (**a**) 1.6 kW, (**b**) 2.0 kW, and (**c**) 2.4 kW.

Figure 12 shows the corrosion morphology of the coating after the potentiodynamic polarization test. The surface of the 1.6 kW coating was pitted and had large corrosion holes (Figure 12a). Corrosion cracks caused by the expansion of corrosion pits appeared in local areas on the surface of the corroded samples, and the formation mechanism was analogous to that of corrosion pits. This behavior was due to the severe segregation of the surface microstructure of the 1.6 kW cladding layer, which had atom-rich and atom-poor regions. During the corrosion process, corrosion was inhomogeneous, and the degree of corrosion in various regions was very inconsistent. Compared with the corrosion morphology of the 1.6 kW coating, the surface corrosion morphology of the 2.0 kW cladding layer did not have large pits and holes due to corrosion. The surface corrosion had a clustered and broken granular morphology, and the overall structure was relatively loose (Figure 12b). Alongside the energy spectrum components in Table 6 (marked points in Figure 12b), the content of B and C in the corrosion hole (Zone 1) of the coating was low, and the area with slight corrosion (Zone 2) contained a large amount of B and C, indicating that carbides and borides could prevent the aggravation of corrosion. The surface corrosion morphology of the 2.4 kW coating was dominated by block-like continuous structures (Figure 12c) with a slight degree of corrosion. The corrosion surface of the coating prepared using 2.4 kW laser power was dominated by sizeable continuous corrosion products (Figure 12c). According to Figure 5, the 2.4 kW coating had less segregation, and the elements were more equally distributed, allowing more uniform resistance to Cl^−^ corrosion. At the same time, the intergranular phase of the 2.4 kW coating was denser than those of the 2.0 and 1.6 kW coatings. This result was consistent with the conclusion in Table 5. The closer the value of *n* (Table 5) was to 1, the more uniform the surface after electrochemical corrosion and the closer the capacitance was to being ideal. The dispersion effect was reduced in the 2.4 kW coating. A summary of the corrosion mechanism is provided in Table 7.

## 4. Conclusions

In this study, a Ni-based alloy coating with a high Ni content was fabricated on a ductile cast iron surface using laser cladding technology with varying levels of laser power. The phase, microstructure, and corrosion properties of the coatings prepared at 1.6, 2.0, and 2.4 kW were studied in detail. The following conclusions can be drawn:

(1) The coatings were mainly composed of solid solution γ-Ni, boride Ni_3_B, silicide Ni_2_Si, and carbide Cr_23_C_6_ phases, which did not change with the variation in the laser power used to prepare the coatings.
(2) The microstructure of the cladding layer crystallized in the shape of dendrites and was mainly composed of a bright white eutectic structure and a dark-gray primary solid solution phase. The eutectic structure mainly occurred between dendrites, and the primary solid solution mainly appeared in the dendrites. With an increase in the laser power, the degree of segregation in the coating decreased, sufficient melting between elements was achieved, and the composition became more uniform. As the laser power increased, more energy was injected into the cladding layer, allowing adequate growth of tissue and a continuous increase in the size of dendrites. The coatings prepared using three levels of laser power showed little change and less dilution at the interface area.
(3) The self-corrosion potential of the 2.4 kW coating was −335.7 mV, and the annual average corrosion rate was 0.00699 mm·a^−^^1^. The coating fabricated at 2.4 kW had the highest corrosion resistance compared with the cladding layers prepared using the two other laser power levels. The surface corrosion of the 2.4 kW coating was mainly in the form of sizeable continuous structures, with a light degree of corrosion and superior corrosion resistance.

## Figures and Tables

**Table 1:** Chemical composition (wt.%) of the ductile cast iron and the Ni-based alloy powder.

Samples	C	Mn	B	Cr	Si	P	S	Fe	Ni
Ductile cast iron	3.72	0.20	―	0.12	2.67	0.05	0.02	Bal	0.47
Ni-based alloy powder	0.08	―	1.36	1.42	2.42	―	―	0.41	Bal

**Table 2:** The main process parameters of Ni-based alloy laser cladding.

Laser Power (kW)	Powder Feeding (r·min^−1^)		Scanning Speed/ (m·s^−1^)	Shielding Gas Flow/(L·s^−1^)	Carrier Gas Flow /(L·s^−1^)	Overlap Rate (%)	LED/ (J·mm^−3^)
1.6 2.0 2.4	2.5		0.03	0.2	0.13	66.7	10.86 13.58 16.30

**Table 3:** EDS elemental content of the points marked in Figure 5.

Laser Power (kW)	EDS Point		Composition (at.%)				
		B	C	Si	Cr	Fe	Ni
1.6	1	1.4	1.5	2.4	0.6	0.3	93.8
	2	10.4	2.8	1.6	0.4	0.1	84.7
2.0	3	2.0	1.8	2.3	0.5	0.2	93.2
	4	6.9	2.1	1.6	0.3	0.1	89.0
2.4	5	1.4	7.3	2.4	0.1	0.3	88.5
	6	6.9	6.6	1.1	0.3	0.3	84.8

**Table 4:** Fitting results of the polarization curve parameters of the cladding layer and substrate.

Samples	*E_corr_* (mV/SCE)	*i_corr_* (µA/cm^2^)	*i_a_* (µA/cm^2^)	*i_c_* (µA/cm^2^)	Corrosion Rate (mm·y^−1^)
1.6 kW	−625.7	8.0440	12.039	8.273	0.12999
2.0 kW	−526.5	3.1560	3.634	4.050	0.03924
2.4 kW	−335.7	0.6763	0.647	0.726	0.00699
Substrate	−713.5	8.535	13.265	8.965	0.46062

**Table 5:** EIS simulated values of cladding layers prepared with different levels of laser power.

Laser Power (kW)	*R*_1_ (Ω·cm^2^)	*R*_2_ (Ω·cm^2^)	CPE_1_	
			*Q*_0_ (s*^n^*/Ω·cm^2^)	*n*
1.6	4.136	2029	9.9965 × 10^−5^	0.829
2.0	6.084	3910	6.5416 × 10^−5^	0.842
2.4	5.619	15268	3.2613 × 10^−5^	0.885

**Table 6:** EDS element content of the points marked in Figure 12b.

EDS Point	Composition (at. %)						
	B	C	Si	Cr	Fe	Ni	O
1	1.1	0.9	0.3	0.2	0.2	96.8	0.4
2	6.8	3.4	0.2	0.1	0.2	88.8	0.4

**Table 7:** Corrosion mechanisms of the cladding layers prepared using different laser powers.

Samples (Coating)	1.6 kW	2.0 kW	2.4 kW
Corrosion degree	Severe	Average	Light
Corrosion morphology	Pits and corrosion holes	Clustered and broken granular	Block-like continuous structure
Results of corrosion	Severe segregation with atom-rich and atom-poor regions	Segregation less than at 1.6 kW	Little segregation and more uniform element distribution

## Data Availability

Not applicable.
